# Protective effects of omega‐3 fatty acids against Alzheimer's disease in rat brain endothelial cells

**DOI:** 10.1002/brb3.1037

**Published:** 2018-10-08

**Authors:** Lijun Wang, Hongguang Fan, Jingchun He, Lifang Wang, Zelong Tian, Chaoran Wang

**Affiliations:** ^1^ Department of Neurology Tianjin Medical University General Hospital Tianjin China; ^2^ Department of Neurology Nankai University Fourth Center Hospital Neurology Center Tianjin China; ^3^ Department of Respiratory Medicine Fourth Central Hospital Affiliated to Nankai University Tianjin China

**Keywords:** Alzheimer's disease, enzymes, lipid peroxidation, omega‐3 fatty acids, rotenone

## Abstract

**Objectives:**

Omega‐3 fatty acids are well‐known unsaturated fatty acids that are essential for growth and development in animals. They primarily participate in the development of intelligence, the nervous system, and vision, and the metabolism of neurotransmitters. Omega‐3 fatty acids have been widely studied in the treatment of Alzheimer's disease (AD). Omega‐3 fatty acids are known to have neuroprotective effects due to their antioxidant capacity. Rotenone has been shown to induce neurotoxicity in vitro.

**Methods:**

We investigated the protective effects of omega‐3 fatty acids against AD in rat brain microvascular endothelial cells (RBMVECs) in vitro. Lipid peroxidation, reactive oxygen species (ROS), glutathione peroxidase (Gpx), reduced glutathione (GSH), superoxide dismutase (SOD), and catalase levels were evaluated in RBMVECs. Flow cytometry was performed to assess apoptosis.

**Results:**

Lipid peroxidation and ROS were reduced in RBMVECs following incubation with omega‐3 fatty acids. Catalase, Gpx, and SOD were increased in RBMVECs following incubation with omega‐3 fatty acids. Flow cytometry showed that incubation with omega‐3 fatty acids reduced the amount of apoptotic RBMVECs.

**Conclusion:**

Our results suggest that omega‐3 fatty acids show potential as a therapeutic agent against AD.

## 1 INTRODUCTION

Alzheimer's disease (AD) is a well‐known chronic neurodegenerative disease characterized by major dementia (Burns & Iliffe, 2009). Short‐term memory loss, language disorientation, loss of motivation, and mood swings are significant symptoms of AD (Burns & Iliffe, 2009). Rotenone is a well‐known pesticide that is present in plants such as the Fabaceae and the jicama vine. It is extensively used to induce experimental models of AD and Parkinson's disease (Caboni et al., 2004; Freestone et al., 2009; Gao, Hong, Zhang, & Liu, 2002; Hartley, Stone, Heron, Cooper, & Schapira, 1994). Synergistic effects are observed when it is used with exogenous factors such as lipopolysaccharide (Gao, Hong, Zhang, & Liu, 2003). Increased production of oxygen free radicals damages cellular macromolecules such as proteins, lipids, and nucleic acids. Elevated levels of reactive oxygen species (ROS) have been associated with aging, diabetes mellitus, Parkinson's disease, and AD (Harman, 1995; Schubert et al., 1995).

Omega‐3 fatty acids are essential unsaturated fatty acids commonly found in food sources such as fish oils and marine animals (Jho, Cole, Lee, & Espat, 2004; Yehuda, Rabinovitz, Crasso, & Mostofsky, 2002). Arachidonic acid and omega‐3 fatty acids are essential components of neuronal membranes and participate in the development of intelligence, the nervous system, and vision, and the metabolism of neurotransmitters (Bakker et al., 2003; Broadhurst et al., 2002). Neurological diseases, memory dysfunction, and loss of learning ability may occur under linolenic acid deficiency (Youdim, Martin, & Joseph, 2000). Consumption of omega‐3 fatty acids during pregnancy reportedly improves the mental capacity of offspring (Helland, Smith, Saarem, Saugstad, & Drevon, 2003). Recommended dietary allowance of omega‐3 fatty acids are 1.6 g/day for men and 1.1 g/day for women. Unsaturated fatty acids are known for their roles in the regulation of cell migration, apoptosis of nerve tissue, and cholinergic, catecholaminergic, and serotonergic synoptic transmissions (Arita et al., 2003; Haag, 2003). Malondialdehyde (MDA), which is produced as an end product of membrane fatty acid oxidation, can further react with proteins to form protein‐aldehyde adducts (Libondi et al., 1994). Omega‐3 fatty acids have been reported to react with aldehydes to protect cellular proteins. This suggests that omega‐3 fatty acids have potential utility as potent therapeutic agents against rotenone‐induced neurotoxicity. In this study, we investigated the in vitro protective effects of omega‐3 fatty acids against AD in rat brain microvascular endothelial cells (RBMVECs).

## 2 MATERIALS AND METHODS

### 2.1 Materials

Omega‐3 fatty acids, fetal bovine serum (FBS), Dulbecco's modified Eagle's medium, sulforhodamine B (SRB), dimethyl sulfoxide, trypsin‐EDTA, 2′,7′‐dichlorofluorescin diacetate (DCFH‐DA) and penicillin‐streptomycin were obtained from Sigma‐Aldrich (Sigma‐Aldrich China, Inc., Shanghai, China). Propidium iodide (PI) and fluorescein diacetate (FDA) were obtained from Santa Cruz Biotechnology, Inc. (Santa Cruz Biotechnology [Shanghai] Co., Ltd., Pudong New District, Shanghai, China).

### 2.2 Cell culture

RBMVECs were obtained from the American Type Culture Collection (ATCC; Manassas, VA). Cells were provided with growth medium containing FBS (10%) and antibiotics (1%) under standard conditions in a CO_2_ incubator (37°C and 5% CO_2_).

### 2.3 Experimental groups

The following experimental treatment groups were used in this study: Group I, sham control; Group II, 5 μM rotenone; Group III, 10 μM rotenone; Group IV, 5 μM rotenone with 5 μM omega‐3 fatty acids; and Group V, 10 μM rotenone with 10 μM omega‐3 fatty acids.

### 2.4 Determination of cell viability

RBMVECs were cultured at a density of 1.7 × 10^4^ cells per well. Cells were treated as described above for 48 hr. After 48 hr of incubation, the medium was carefully removed and cells were washed twice with phosphate buffered saline (PBS) before the plates were fixed with 70% acetone. SRB assays were performed on the fixed plates. In brief, 100 μl of SRB reagent (0.4 mg/L) was added to the dried wells of a 96‐well plate, then the plate was incubated at room temperature for 12 hr. After incubation, the SRB reagent was decanted and the plate was washed three times with 1% acetic acid. The plate was dried at 60°C, and then, cell morphology was observed under a reflected light microscope. Images were taken and cell viability was compared. The 96‐well plates containing the cells were treated with 10 mM Tris base for 4 hr. Spectral data were collected under a 96‐well plate reader at 510 nm to calculate the inhibition concentration (Nagajyothi, Muthuraman, Sreekanth, Kim, & Shim, 2017).

### 2.5 Determination of lipid peroxidation

RBMVECs were cultured at a density of 1.7 × 10^4^ cells per well. Cells were treated as described above for 48 hr. The medium was removed after incubation and cells were washed twice with PBS. The MDA content was determined by measuring the thiobarbituric acid reactive species. The final product was measured at 534 nm (Muthuraman, Muthuviveganandavel, & Kim, 2015).

### 2.6 Determination of reduced glutathione

RBMVECs were cultured at a density of 1.7 × 10^4^ cells per well. Cells were treated as described above for 48 hr. The medium was removed after incubation and cells were washed twice with PBS. Reduced glutathione (GSH) content was determined by measuring the final product at 405 nm (Muthuraman et al., 2015).

### 2.7 Determination of ROS levels

RBMVECs were cultured at a density of 1.7 × 10^4^ cells per well. Cells were treated as described above for 48 hr. The medium was removed after incubation and cells were washed with PBS. The cells were incubated with DCFH‐DA for 30 min, and ROS levels were measured under a fluorescence plate reader (Muthuraman, Kim, Muthuviveganandavel, Vikramathithan, & Ravikumar, 2016).

### 2.8 Determination of glutathione peroxidase

RBMVECs were cultured at a density of 1.7 × 10^4^ cells per well. Cells were treated as described above for 48 hr. The medium was removed after incubation and cells were washed with PBS. Glutathione peroxidase (Gpx) was measured using spectrophotometry (Muthuraman et al., 2015).

### 2.9 Measurement of antioxidant enzyme activities

RBMVECs were cultured at a density of 1.7 × 10^4^ cells per well. Cells were treated as described above for 48 hr. The medium was removed after incubation and cells were washed with PBS. Superoxide dismutase (SOD) and catalase enzyme activities were measured using spectrophotometry (Muthuraman et al., 2015).

### 2.10 Flow cytometric analysis of apoptosis

RBMVECs were cultured at a density of 1.7 × 10^4^ cells per well. Cells were treated as described above for 48 hr. The medium was removed after incubation and cells were washed with PBS. The percentages of apoptotic and non‐apoptotic cells were determined using PI and FDA in fluorescence‐activated cell sorting (Muthuraman, Enkhtaivan, & Kim, 2016).

### 2.11 Mitochondrial fragmentation assay

RBMVECs were cultured at a density of 1.7 × 10^4^ cells per well. Cells were treated as described above for 48 hr. The medium was removed after incubation and cells were washed with PBS. Cells were incubated with MitoTracker Red (40 nM) for 30 min and with Hoechst 33258 (2 μg/ml) for 15 min to stain mitochondria and nuclei, respectively (Muthuraman, Mistry, Enkhataivan, & Kim, 2016).

### 2.12 Terminal deoxynucleotidyl transferase dUTP nick end labeling (TUNEL) assay

RBMVECs were cultured at a density of 1.7 × 10^4^ cells per well. Cells were treated as described above for 48 hr. The medium was removed after incubation and cells were washed with PBS. Cells were incubated with terminal deoxynucleotidyl transferase (TdT) and stained with PI to visualize DNA damage (Muthuraman, Mistry, et al., 2016).

### 2.13 Statistical analysis

Experimental values are expressed as the mean ± standard error of the mean (*SEM*). The differences between treatment and control groups were determined using analysis of variance (ANOVA) followed by Student's *t* test. *p*‐Values < 0.05 were considered statistically significant.

## 3 RESULTS

### 3.1 Effects of omega‐3 fatty acids on RBMVEC viability

**Figure 1:**
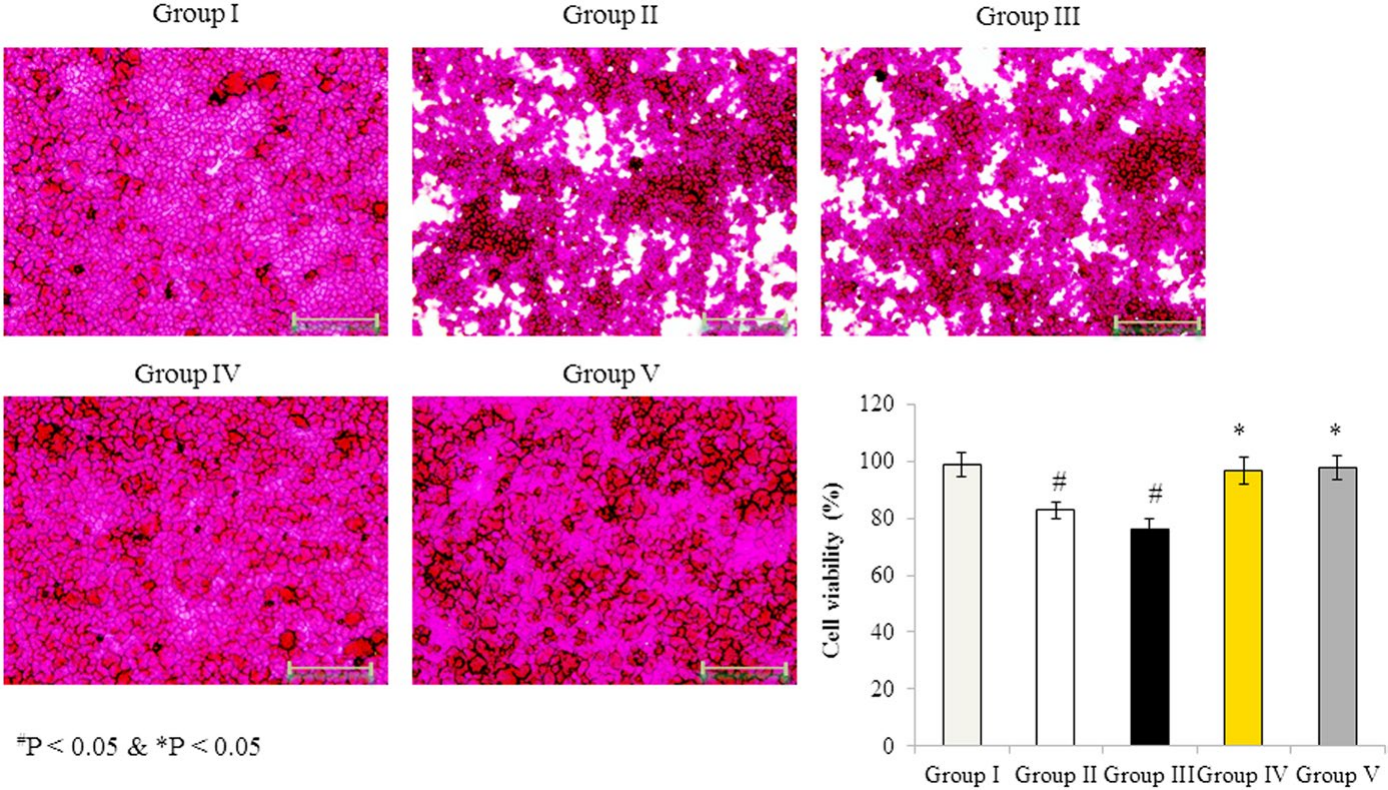
Effects of omega‐3 fatty acids on rat brain microvascular endothelial cell viability. Cells were treated as described in Methods section. Values are expressed as a percentage of cell viability. ^#^ *p *<* *0.05 versus Groups II & III; *p <* *0.05 versus Groups IV & V. Scale bar = 30 μm

The protective effects of omega‐3 fatty acids on rotenone‐induced cell viability were determined. The viability of RBMVECs was reduced by 16.15% and 29.38% in the rotenone‐treated Groups II and III, respectively. However, no significant growth inhibition was observed in cells incubated with both rotenone and omega‐3 fatty acids (Groups IV and V) (Figure 1, *p* < 0.05).

### 3.2 Effects of omega‐3 fatty acids on lipid peroxidation in RBMVECs

**Figure 2:**
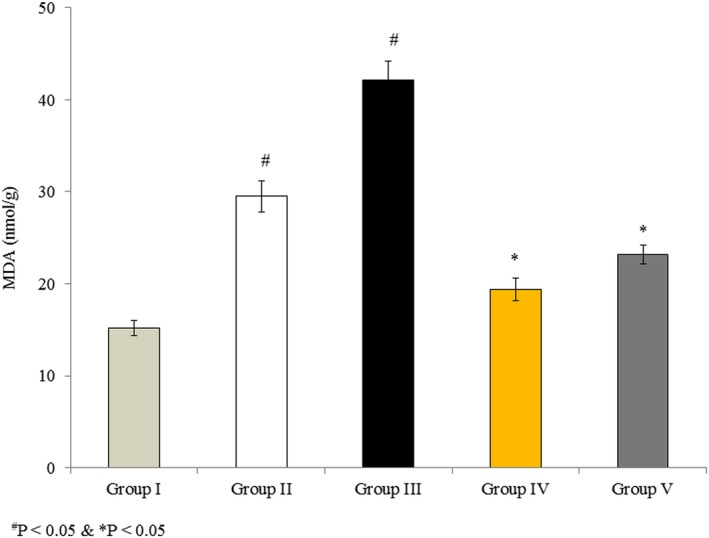
Omega‐3 fatty acids attenuate lipid peroxidation in rat brain microvascular endothelial cells. Cells were treated as described in Methods section. Values are expressed as nmol/g. ^#^ *p *<* *0.05 versus Groups II & III; *p <* *0.05 versus Groups IV & V

Malondialdehyde was measured as the end product of lipid peroxidation. MDA content was 15.16 nmol/g in untreated cells (Group I), whereas it dramatically increased by 94.6% and 178% in Group II and Group III, respectively. However, MDA levels increased by only 28% and 52.7% in Groups IV and V, respectively, indicating that co‐treatment with omega‐3 fatty acids attenuated the rotenone‐induced elevation in lipid peroxidation (Figure 2, *p* < 0.05).

### 3.3 Effects of omega‐3 fatty acids on GSH content in RBMVECs

**Figure 3:**
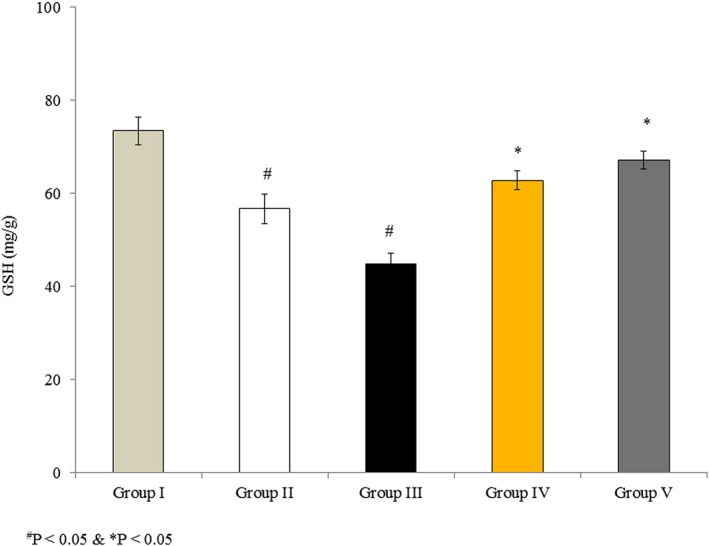
Effects of omega‐3 fatty acids on glutathione (GSH) in rat brain microvascular endothelial cells. Cells were treated as described in Methods section. Values are expressed as mg/g. ^#^ *p *<* *0.05 versus Groups II & III; *p <* *0.05 versus Groups IV & V

Glutathione is a vital antioxidant that provides reducing equivalents for Gpx. GSH content was 73. 42 mg/g in untreated cells (Group I), and it decreased dramatically by 22.7% and 39% in the rotenone‐treated Groups II and III, respectively. However, this decrease in GSH content was reduced in cells incubated with rotenone and omega‐3 fatty acids together, to 14.4% and 8.5% in Groups IV and V, respectively (Figure 3, *p* < 0.05).

### 3.4 Effects of omega‐3 fatty acids on ROS in RBMVECs

**Figure 4:**
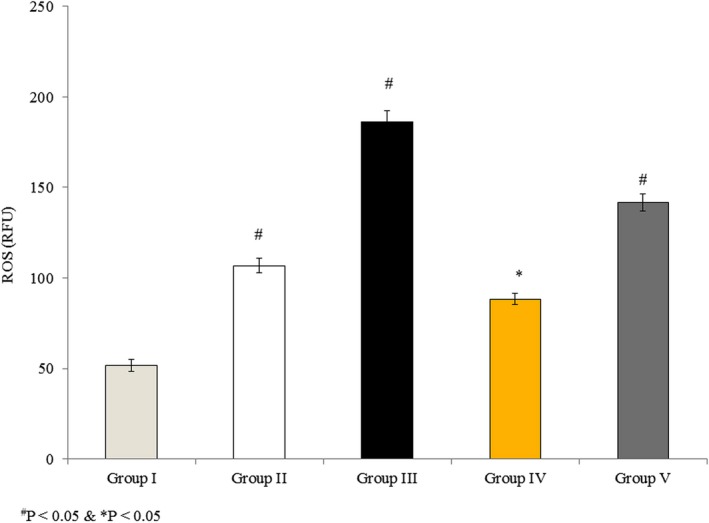
Omega‐3 fatty acids attenuate the generation of reactive oxygen species (ROS) in rat brain microvascular endothelial cells. Cells were treated as described in Methods section. Values are expressed as relative fluorescence units (RFU). ^#^ *p *<* *0.05 versus Groups II & III; *p <* *0.05 versus Groups IV & V

Reactive oxygen species are produced by cells during mitochondrial metabolism and in response to xenobiotics. ROS content was 51.62 relative fluorescence units in untreated cells (Group I), and it significantly increased by 106.7% and 260.8% in Groups II and III, respectively, following rotenone treatment. ROS levels only increased by 71.2% and 174.6% in Groups IV and V, respectively, which were treated with both rotenone and omega‐3 fatty acids (Figure 4, *p* < 0.05).

### 3.5 Effects of omega‐3 fatty acids on Gpx activity in RBMVECs

**Figure 5:**
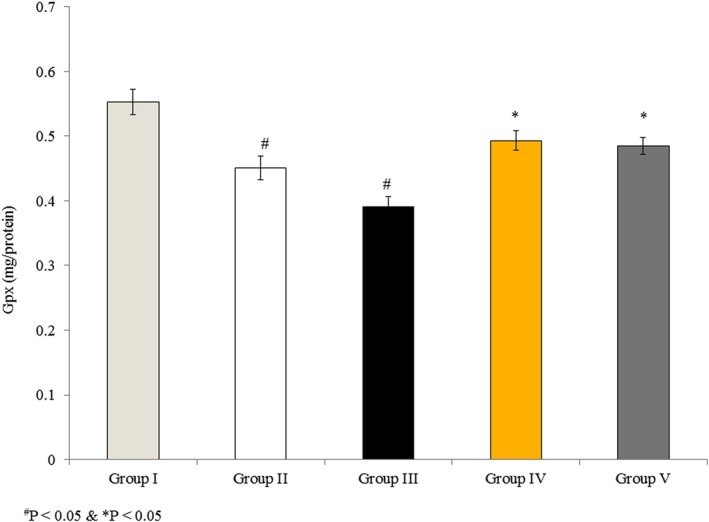
Effects of omega‐3 fatty acids on glutathione peroxidase (Gpx) activity in rat brain microvascular endothelial cells. Cells were treated as described in Methods section. Values are expressed as mg/protein. ^#^ *p *<* *0.05 versus Groups II & III; *p <* *0.05 versus Groups IV & V

Glutathione peroxidase is a vital antioxidant enzyme that reduces lipid hydroxides with the help of GSH. Gpx activity was 0.553 mg/protein in untreated cells (Group I), and it decreased by 18.4% and 29.2% in Groups II and III, respectively, following rotenone treatment. Cells incubated with rotenone and omega‐3 fatty acids together showed less reduction in Gpx activity, 10.4% and 12.3% in Groups IV and V, respectively (Figure 5, *p* < 0.05).

### 3.6 Effects of omega‐3 fatty acids on SOD and catalase activities in RBMVECs

**Figure 6:**
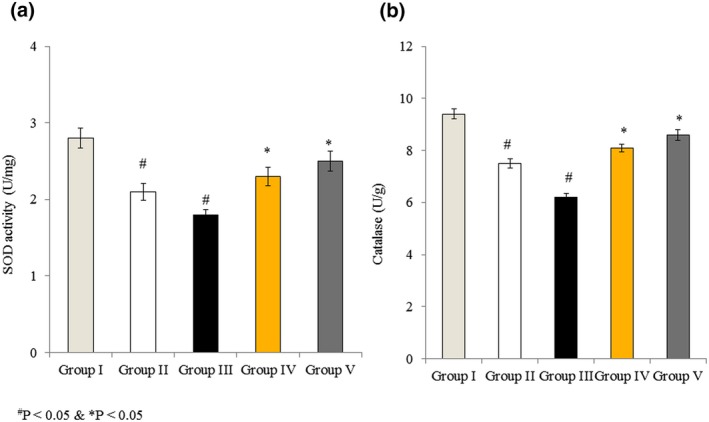
Effects of omega‐3 fatty acids on superoxide dismutase (SOD) and catalase activity in rat brain microvascular endothelial cells. Cells were treated as described in Methods section. (a) SOD activity is expressed as U/mg. (b) Catalase activity is expressed as U/g. ^#^ *p *<* *0.05 versus Groups II & III; *p <* *0.05 versus Groups IV & V

In untreated cells (Group I), the SOD and catalase activities were 2.8 and 9.4 U/g, respectively. SOD activity was reduced by 25% and 35.7% in Groups II and III, respectively, whereas the cells incubated with both rotenone and omega‐3 fatty acids showed less reduction in SOD activity, 17.9% and 10.7% in Groups IV and V, respectively (Figure 6a, *p* < 0.05). Catalase activity was reduced by 20.2% and 34% in Groups II and III, respectively, whereas cells incubated with both rotenone and omega‐3 fatty acids exhibited less reduction in catalase activity, 13.8% and 8.5% in Groups IV and V, respectively (Figure 6b, *p* < 0.05).

### 3.7 Effects of omega‐3 fatty acids on apoptosis in RBMVECs

**Figure 7:**
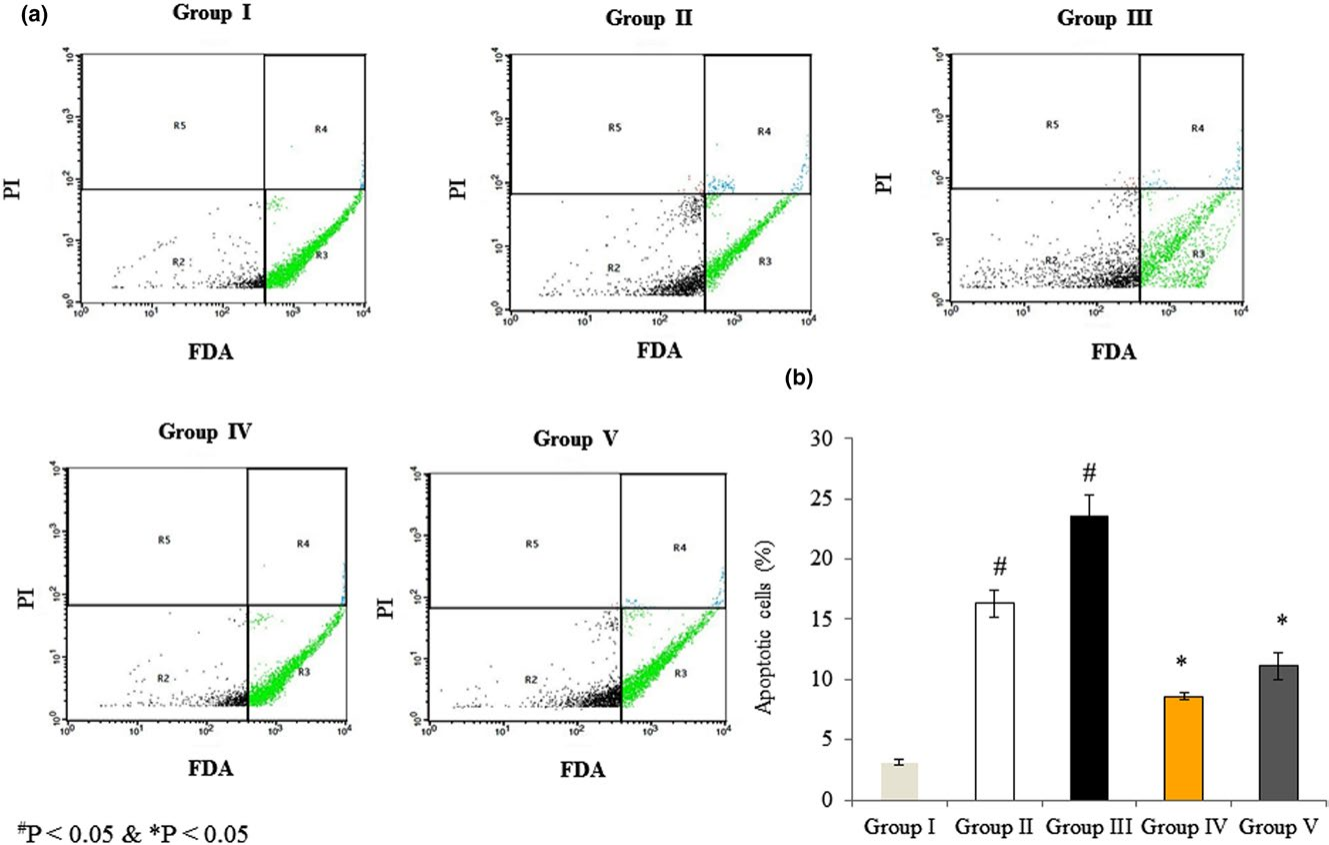
Effects of omega‐3 fatty acids on apoptosis in rat brain microvascular endothelial cells as assessed by flow cytometry. Cells were treated as described in Methods section. Fluorescein diacetate (FDA) and propidium iodide (PI) were used to distinguish viable and apoptotic cells. (a) R2: FDA and PI negative; R3: FDA positive; R4: FDA and PI positive; and R5: PI positive. (b) The percentage of apoptotic cells (%). ^#^ *p *<* *0.05 versus Groups II & III; *p <* *0.05 versus Groups IV & V

Fluorescein diacetate and PI were used to differentiate apoptotic from non‐apoptotic cells. There was a large number of active, non‐apoptotic RBMVECs in the control group (>96%), whereas apoptotic cell numbers increased by 16.32% and 23.52% in Groups II and III, respectively. However, the rotenone‐induced increase in apoptosis was attenuated by co‐treatment with omega‐3 fatty acids to 8.62% and 11.12% in Groups IV and V, respectively (Figure 7, *p* < 0.05).

### 3.8 Effects of omega‐3 fatty acids on mitochondrial morphology

**Figure 8:**
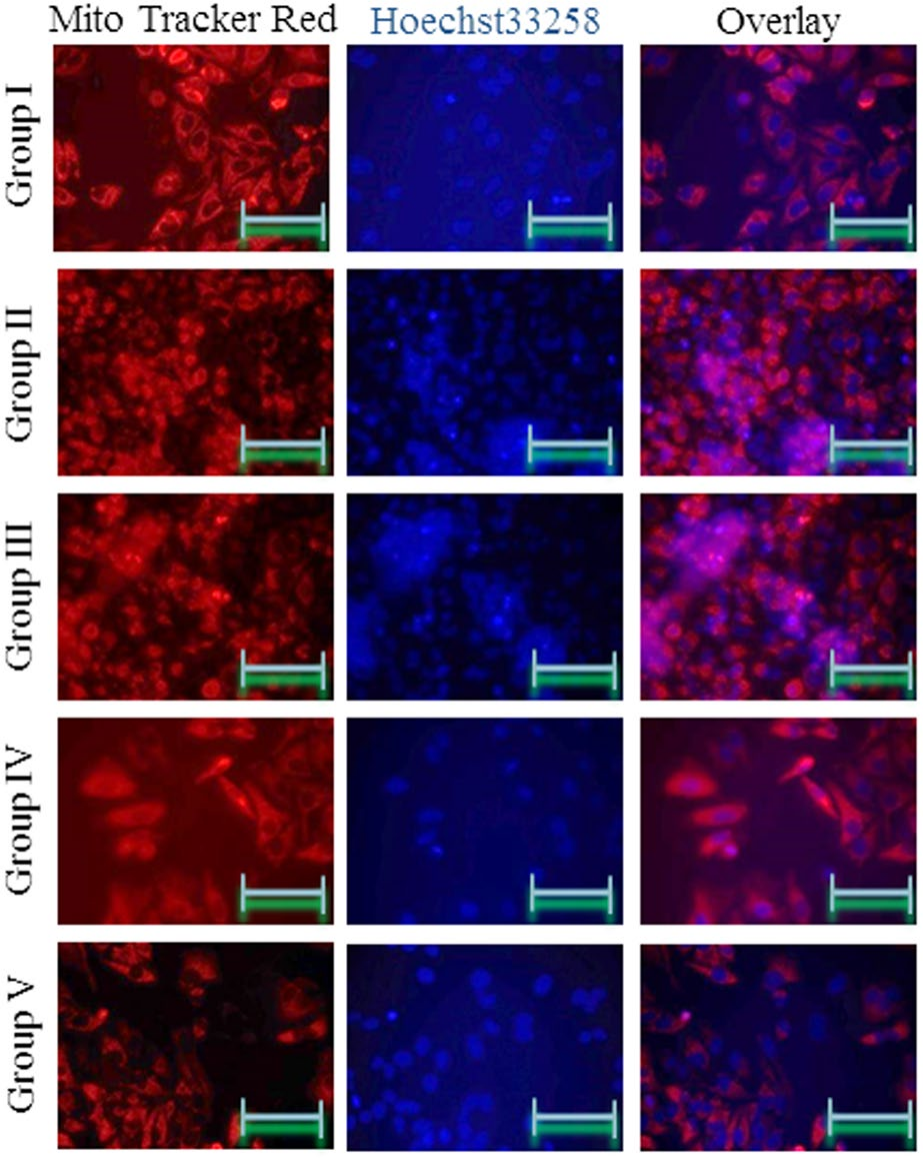
Omega‐3 fatty acids attenuate mitochondrial damage in rat brain microvascular endothelial cells. Cells were treated as described in Methods section. Cells were stained with MitoTracker Red and Hoechst 33258 to distinguish mitochondria and nuclei. Scale bars = 50 μm

MitoTracker Red and Hoechst 33258 were used to stain mitochondria and nuclei, respectively, to assess cell morphology following treatment. Sham control cells showed normal mitochondrial and nuclear morphology. Clumped and shrinked mitochondria were predominantly observed in Groups II and III. The cells incubated with both rotenone and omega‐3 fatty acids (Groups IV and V) showed less mitochondrial and nuclear damage than those incubated with rotenone alone (Figure 8).

### 3.9 Effects of omega‐3 fatty acids on DNA damage

**Figure 9:**
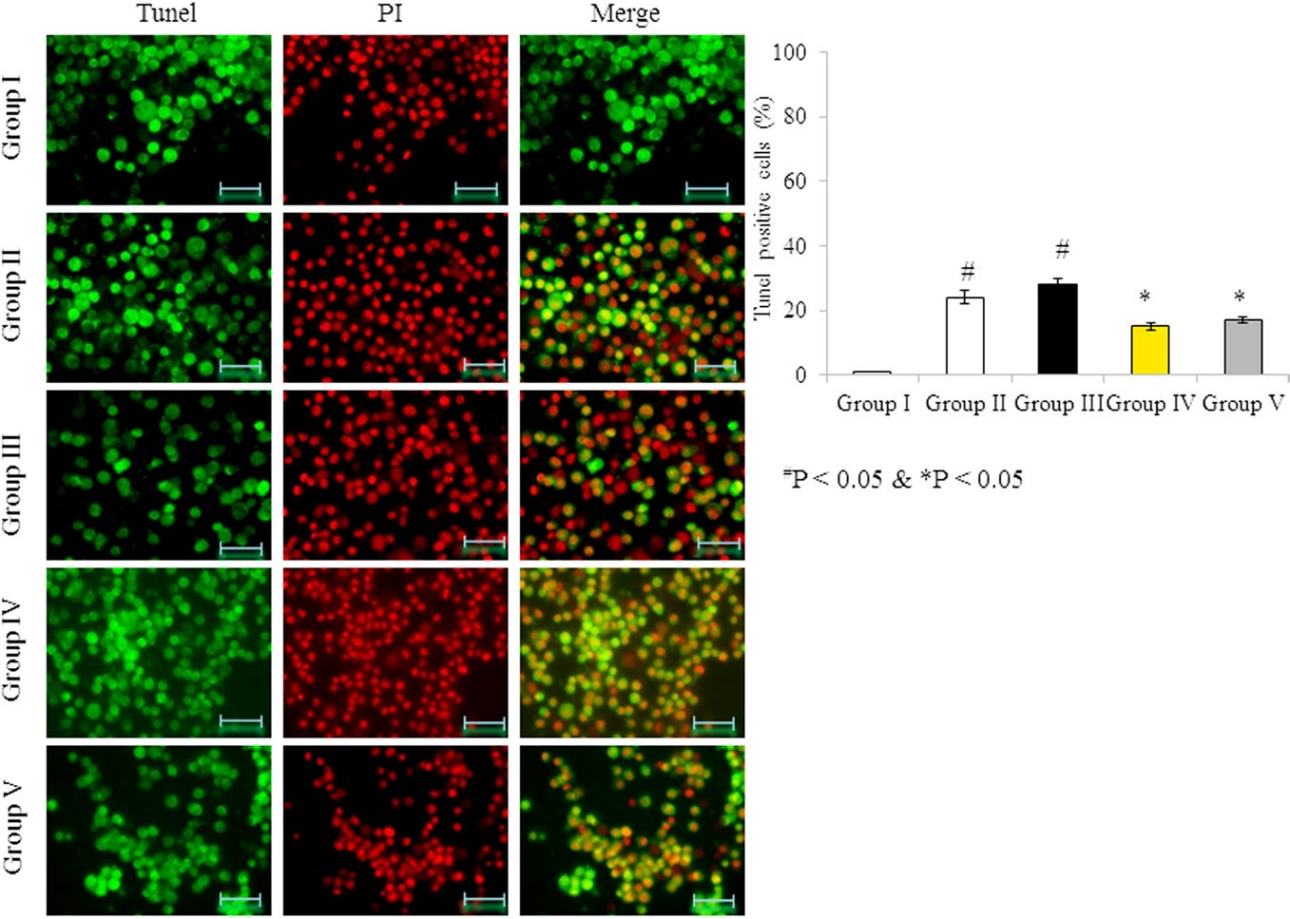
Omega‐3 fatty acids attenuate DNA damage in rat brain microvascular endothelial cells. Cells were treated as described in Methods section. (a) Cells were stained with fluorescein isothiocyanate (FITC) and propidium iodide to localize DNA damage. (b) The percentage of terminal deoxynucleotidyl transferase dUTP nick end labeling (TUNEL)‐positive cells (%). Scale bar = 50 μm

Fluorescein isothiocyanate and PI were used to assess damaged and viable DNA in RBMVECs following treatment. Sham control cells exhibited normal, viable DNA, and no visible DNA damage was observed. The number of TUNEL‐positive cells (indicative of damaged DNA) increased by 24% and 28% in the rotenone‐treated Groups II and III, respectively. However, the cells incubated with both rotenone and omega‐3 fatty acids exhibited fewer TUNEL‐positive cells, only 15% and 17% in Groups IV and V, respectively (Figure 9, *p* < 0.05).

## 4 DISCUSSION

We observed the protective effects of omega‐3 fatty acids against AD in rat brain endothelial cells in vitro. Structural and morphological changes are considered critical factors in apoptosis. A good anti‐cancer therapeutic should induce apoptosis in tumor cells (Frankfurt & Krishan, 2003). Assessing the viability and morphology of RBMVECs is useful in analyzing the protective effects of omega‐3 fatty acids against AD. Accelerated oxidative damage is a critical factor in AD, and the products of oxidative damage appear in the early clinical stages of the disease (Markesbery, Kryscio, Lovell, & Morrow, 2005; Montine et al., 2002; Querfurth & LaFerla, 2010). Membrane fatty acid oxidation occurs due to increased production of free radicals. An imbalance between cellular antioxidant mechanisms and rate of free radical production induces oxidative stress (Querfurth & LaFerla, 2010).

Glycated proteins accumulate during the normal aging process in rats, whereas higher amounts are found in AD (Smith et al., 1995, 1996; Stadtman, 1992). In our study, omega‐3 fatty acids reduced lipid peroxidation in RBMVECs, indicating that omega‐3 fatty acids are protective against AD. Rotenone inhibits the electron transport chain (Storch, Kaftan, Burkhardt, & Schwarz, 2000), and it exerts neurotoxicity through increased production of ROS and apoptosis. Omega‐3 fatty acid consumption has been associated with a reduced risk of several neurological diseases, and these fatty acids are essential for maintaining the structure and fluidity of neural membranes (Salvati, Attorri, Di Benedetto, Di Biase, & Leonardi, 2006). Treatment with polyunsaturated fatty acids has been reported to reduce brain infarct size in a rat model of ischemia/reperfusion (I/R) injury (Choikwon, 2004).

Catalase, SOD, and Gpx are key enzymes in the alleviation of oxidative stress. Avramovic et al. (2012) reported increased SOD activity and reduced lipid peroxidation following omega‐3 fatty acid supplementation in rats. As neural tissues are more susceptible to lipid peroxidation, beneficial health effects are observed in these tissues following fish oil treatment (Bas et al., 2007). Fish oil treatment reduced the level of lipid peroxidation in I/R rats (Ozen et al., 2008). Neuroprotection has been reported in rat hippocampus following omega‐3 fatty acid supplementation. These findings support our results that lipid peroxidation and ROS generation were reduced, whereas GSH, SOD, catalase, and Gpx were increased, in RBMVECs following omega‐3 fatty acid supplementation.

## 5 CONCLUSION

Supplementation with omega‐3 fatty acids reduced lipid peroxidation and ROS generation and increased the levels of GSH, SOD, catalase, and Gpx in RBMVECs. This suggests that omega‐3 fatty acids are a potential therapeutic agent against AD.
